# “They Wait until the Disease Has Taking over You and the Doctors Cannot Do Anything about It”: Qualitative Insights from Harambee! 2.0

**DOI:** 10.3390/ijerph182312706

**Published:** 2021-12-02

**Authors:** Shukri A. Hassan, Farah Mohamed, Najma Sheikh, Guiomar Basualdo, Nahom A. Daniel, Rahel Schwartz, Beyene Tewelde Gebreselassie, Yikealo K. Beyene, Luwam Gabreselassie, Kifleyesus Bayru, Bethel Tadesse, Hirut Amsalu Libneh, Mohamed Shidane, Sophia Benalfew, Ahmed Ali, Deepa Rao, Rena C. Patel, Roxanne P. Kerani

**Affiliations:** 1 Department of Medicine, University of Washington, Seattle, WA 98104, USA; rcpatel@uw.edu (R.C.P.); rkerani@uw.edu (R.P.K.); 2 Somali Health Board, Tukwila, WA 98188, USA; shidane@somalihealthboard.org (M.S.); ahmed.abdille@gmail.com (A.A.); 3 Department of Global Health, University of Washington, Seattle, WA 98195, USA; nmsheikh@uw.edu (N.S.); deeparao@uw.edu (D.R.); 4 Department of Anthropology, University of Washington, Seattle, WA 98195, USA; grb08@uw.edu; 5 Department of Biology, University of Washington, Seattle, WA 98195, USA; nahomd@uw.edu; 6 Ethiopian Community Center in Seattle, Seattle, WA 98118, USA; rschwartz@seattleymca.org (R.S.); b_belay@yahoo.com (B.T.); Mlibneh@gmail.com (H.A.L.); sophiab@ecseattle.org (S.B.); 7 Ethiopian Health Board, Seattle, WA 98118, USA; 8 Eritrean Health Board, Seattle, WA 98122, USA; betewelde@yahoo.com (B.T.G.); beyikealo@gmail.com (Y.K.B.); luwamg3@gmail.com (L.G.); kifleyesusb@lifelong.org (K.B.)

**Keywords:** African immigrant, preventive healthcare, healthcare access, immigrant, screening, prevention, migrant, culture, religion

## Abstract

African immigrants make up a large subgroup of Black/African-Americans in the US. However, because African immigrant groups are typically categorized as “Black,” little is known about their preventative healthcare needs. Differences in culture, life and healthcare experiences between African immigrant populations and US-born people may influence preventive health care uptake. Thus, policymakers and healthcare providers lack information needed to make informed decisions around preventive care for African immigrants. This formative study was conducted among the largest East African immigrant communities in King County, WA. We recruited religious leaders, community leaders, health professionals, and lay community members to participate in thirty key informant interviews and five focus group discussions (*n* = 72 total), to better understand preventative healthcare attitudes in these communities. Through inductive coding and thematic analysis, we identified factors that impact preventative healthcare attitudes of the Somali, Ethiopian and Eritrean immigrant communities and deter them from accessing and utilizing healthcare. Cultural beliefs and attitudes around preventative healthcare, mistrust of westernized healthcare, religious beliefs/views, intersecting identities and shared immigrant experiences all influence how participants view preventative healthcare. Our results suggest that interventions that address these factors are needed to most effectively increase uptake of preventative healthcare in African immigrant communities.

## 1. Introduction

African immigrants represent a small but growing share of the American population, increasing 251% from 2000 to 2016. East African immigrants represent 36% of the total Sub-Saharan African immigrant population in the U.S., and constitute the largest proportion of African immigrants in King County, WA [1]. Immigrants generally arrive in the U.S. in better health than their U.S.-born counterparts—what is known as the “healthy immigrant effect”—thought to be related to healthier pre-immigration behaviors, including diet and physical activity levels, and the impact of younger, healthier people, possibly with higher socioeconomic status, being more able and likely to migrate [2,3]. In fact, African immigrants have better health upon arrival in the U.S. than immigrants from most other regions of the world [2]. Studies have found that African immigrants, one of the fastest growing immigrant groups in the U.S., have lower rates of hypertension [4,5,6], diabetes [4,6,7], and obesity [4,7,8] than those born in the U.S. Several explanations have been offered for such observations, including the acculturation theory, according to which recent immigrants assimilate aspects of the western lifestyle and thus are exposed to harmful environmental factors [9]. However, there is a growing body of evidence that African immigrants have lower uptake of preventative health services than U.S.-born people, including for vaccinations [10,11], cancer screening [10,12,13,14], and HIV testing [15]. Consequently, African immigrant communities are disproportionately impacted by late diagnoses across a broad range of health conditions, including some cancers [16], HIV [17,18], and hepatitis B [16].

Immigrants face a variety of barriers to successfully accessing a broad range of healthcare in the U.S. Many of these barriers are structural in nature, such as lower rates of insurance coverage, cost, discrimination, language barriers, and lower health literacy among immigrants vs. non-immigrants, often compounded by inexperience among immigrants with the U.S. healthcare system [4,12,19,20,21,22,23,24]. Some immigrants also fear that using publicly-funded health care services or insurance might negatively impact immigration proceedings [25,26].

However, the uptake of health care by individual immigrant communities likely also varies by community and cultural norms, including attitudes around health, wellness, and medicine [27,28,29,30]. In our previous formative work examining barriers to HIV testing in largely East African immigrant communities in the Seattle area, participants reported that uptake of preventative services generally was low in their communities [31]. Indeed, in the same study, we found a high prevalence of non-communicable diseases among African immigrant participants in community-based health fairs, including overweight/obesity, hypercholesterolemia, and hypertension [32]. As a result, in our current study to evaluate the effect of HIV-related and intersectional stigma on HIV testing behaviors, we sought to better understand more generally preventative health attitudes among three East African immigrant communities in Seattle. We anticipated that stigmas would not be limited to HIV only and would apply to other health conditions as well. In our study we define preventative health as health screenings and routine medical visits that are performed in order to prevent or detect serious illnesses.

## 2. Materials and Methods

### 2.1. Study Setting, Academic-Community Partnership, and Positionality

This study was part of an academia-community partnership between the University of Washington (UW) researchers and the Somali, Ethiopian and Eritrean health organizations in King County, WA [31,32]. This partnership arose from our ongoing work to address barriers to HIV testing amongst local African immigrant communities utilizing the principles of community-based participatory research. We prioritized principles of equity (e.g., equitable distribution of financial resources), justice (e.g., representation of community partners in all steps of the research process), and sustainability (e.g., bidirectional capacity-building and commitment to organizational priorities) in building this partnership.

The project team consisted of three primary investigators, two of which belonged to South Asian immigrant communities, the community partners, study staff, three undergraduate research assistants all belonging to local East African immigrant communities, and one undergraduate research assistant who belonged to a Latinx immigrant community. All investigators considered how their positionalities differed from participant experiences, and thus, took a learning perspective to interactions with participants in order to minimize bias. All members of the project team met regularly during project planning stages, implementation, analysis, and community dissemination.

### 2.2. Data Collection

Overall approach: We used the qualitative descriptive method, as described by Sandelowski [33,34], which included conducting both key informant interviews (KIIs) and focus group discussions (FGDs). In order to prepare for our larger FGDs, we first conducted the KIIs from October 2019 to January 2020. This method allowed us to use the preliminary analyses of the KIIs to inform our focus group discussion guides and interviews from March to late April 2020. All interviews were conducted in King County, WA and the KIIs took place in person before the start of the COVID-19 pandemic, whereas the FGDS occurred virtually during the pandemic.

Sampling: Through the utilization of purposeful sampling, we identified categories of key informants/participants to include in the KIIs and focus groups, and from there our community partners chose potential study participants and the manner in which they recruited them. For the KIIs, we recruited and interviewed lay community members, healthcare professionals, people living with HIV (PLWHIV), and religious leaders who belong to the East African immigrant community. We recruited from these groups because our community partner organizations believed their opinions were critical in informing our work on HIV testing and preventative health care. We oversampled religious leaders for the FGDs, because KII interview participants felt that religious leaders were key gatekeepers that would be important to the success of a potential HIV stigma reduction intervention. We used case management organizations and provider referrals from the UW-affiliated county hospital HIV clinic to recruit the PLWHIV participants. To avoid any potential traumatizing conversations or inadvertent disclosure of HIV status, we did not attempt to recruit PLWHIV for the focus group discussions. The Human Subjects Division of the University of Washington approved this study, and we obtained oral informed consent in participants’ chosen language. We collected patient demographics from every participant (such as gender, age, country of birth, religious affiliation and occupation), and they each received a cash reimbursement of $50 for their participation in either the KII or FGD. Our sampling numbers were guided by saturation of themes within the categories of the participants.

Interviewers and facilitators: Community partners selected the facilitators and interviewers from within their respective communities. Each interviewer/facilitator were bi- or trilingual and first generation Americans speaking at least two of the following languages: Somali, Amharic, Tigrinya, Swahili and English. Although the majority of the interviewers indicated they had experience conducting KIIs and all the facilitators had prior experience conducting FGDs, we held centralized trainings in October 2019 for interviewers and February 2020 for the facilitators. Interviews were conducted in whichever language matched the participants’ language preference whenever feasible. However, related to concerns about potentially disclosing participant’s HIV status, PLWHIV were given the option to interview with project members belonging to any of the African immigrant communities or U.S.-born study staff. Two participants chose to hold such interviews with staff outside of their own communities and consent was given in English. In the FGDs, the facilitator and note taker conducted the discussions within their respective community, and three study team members (RCP, RPK, and FM) joined at the beginning of every FGD to give thanks and welcome the participants in English. The facilitator provided live interpretation of these remarks and the remainder of the discussion would proceed with members of the respective community and in their chosen language. Our Eritrean community partner chose to conduct one FGD that was not gender-specific, whereas our Somali and Ethiopian community partners conducted four gender-specific FGDs. The Eritrean team suggested that male and female participants will freely engage and contribute to the discussions, however, the other two groups thought gender-specific discussions will be culturally appropriate and reduce barriers for female participants.

Interview/discussion procedures: The key informant interviews were held across the greater metropolitan Seattle area to accommodate the preferences of the interviewer and interviewee. Locations such as health organizations offices and ethnic community centers served as meeting spaces to hold KIIs. Due to the COVID-19 pandemic, FGDs were conducted virtually through Zoom. Each KII and FGD were audio recorded to then be translated and transcribed by the facilitator or another team member of the same community partnership. The interviewer reviewed all English translations/transcriptions for accuracy, and if there were any discrepancies they were resolved between the two team members.

Interview/discussion guides: The KII guide focused on the following five main domains: (1) interactions with the U.S. healthcare system; (2) barriers to health screenings; (3) stigma around health screenings and HIV-related stigma, including for HIV testing; (4) intersectional stigma and influence on HIV testing; and (5) how to reduce stigma around health screenings, including HIV testing. The FGD guide focused on the following three main domains: (1) stigma around health screenings and HIV-related stigma, including for HIV testing; (2) intersectional stigma and influence on HIV testing; and (3) intervention development for stigma reduction around HIV, with emphasis on the role of religious leaders and institutions.

Our initial development of the KII and FGD guides were informed by Earnshaw’s Stigma and HIV Disparities Model, Rao et al.’s multilevel HIV stigma model, and the socioecological model [35,36,37]. While Earnshaw and colleagues developed the Stigma and HIV Disparities Model to demonstrate the concurrent impact of coexisting marginalized identities and to explain how intersectional stigma has a negative influence on HIV outcomes, many of these same factors, including structural racism and discrimination related to being an immigrant, also impact general preventative health outcomes. Similarly, we also drew on the multilevel HIV stigma model developed by Rao et al. and the socioecological model when developing our interview guides, and often generalized on the constructs to include general preventative health. The KII and FGD guides are included as Supplementary Materials.

### 2.3. Data Analysis

To analyze the qualitative data, the KII and FGD English transcripts were uploaded to NVivo (version 12.0, QRS International Pty Ltd., Melbourne, Australia). The research assistants (SAH, NJ, GB) were guided by team members RCP, FM, and RPK in coding the transcripts and utilizing inductive coding techniques. KII and FGD guides were used to create the initial codebook along with team members’ input after they read some of the initial transcripts. The codebook has since been adapted by team members as they progressed in coding all the transcripts. Three team members coded the first initial transcript together and then double-coded 1–2 additional transcripts; all discrepancies in coding were resolved through team meetings. Remaining transcripts were individually coded by the research assistants, then one coder (SAH) reviewed coding for all transcripts. Through several team meetings, we used thematic analysis and inductive coding techniques to arrange all codes into three overarching thematic domains, their corresponding sub-themes that were both divergent and converged, along with quotes to illustrate depth and meaning [38,39].

## 3. Results

A total of 72 participants (Table 1) contributed to thirty KIIs and five FGDs. Twenty-six (36%) of the 72 total participants were born in Ethiopia, 27(37%) were born in Somalia, and 17 (23%) were born in Eritrea. One participant was born in Kenya, and one of the participants identifying with the Eritrean community was born in the U.S. Thirty-five (49%) participants identified as female and 37 (51%) identified as males. Participant ages ranged from 22–67 years, with the majority being between 30 to 49 years. Participants primarily worked as religious leaders (*n* = 14) or as healthcare professionals (*n* = 15). Five individuals identified as PLWHIV, and all were KII participants.

Overall, four major themes that influence preventative health care attitudes of the Somali, Ethiopian, and Eritrean immigrant communities in the Seattle area emerged from our findings: (1) culture beliefs and attitudes in shaping community views of healthcare, (2) religious beliefs/views on manifestation of illness (3) immigrant shared experiences, and (4) structural barriers related to health systems. We discuss these themes in further detail below, beginning with a broad description of cultural beliefs related to preventative health care in these communities to provide greater context for our later findings. In general, there were far more commonalities across the three communities than differences, and we highlight where differences exist; otherwise, the findings are applicable to all three communities. In addition to quotations included in-text, Table 2 provides supporting quotations from the interviews and focus groups, organized by the major themes described below.

### 3.1. Culture Beliefs and Attitudes in Shaping Communities View of Healthcare

#### 3.1.1. Physical Manifestations of Illness

In each of the three country-of-origin communities, participants agreed that seeking health care is undertaken primarily in two situations: (1) when having physical manifestations of illness, or (2) when illness interferes with day-to-day activities. Lack of physical manifestations or symptoms relating to illness results in delays in seeking medical attention and lessens any motivations to prevent or diagnose early. *“In our culture, we go to a clinic/hospital only if one is very sick. Even when we’re sick, including myself, the illness must interfere with your daily life for you to see a doctor. Otherwise, if you can bear the pain while working and doing your daily life, we don’t bother to see a doctor.”* (50 year old, male, Eritrean, nurse). Health care is only sought when the health condition has progressed too far to ignore, it becomes visibly evident that the individual is ill, and/or when it interferes with daily activities.

#### 3.1.2. Fear of Being Ill

Several participants stated that their reasoning for not using preventative health care was due to their larger fear of being ill, as well as a linked fear that a diagnosis of an illness or condition would result in death. Participants stated that by forsaking preventative healthcare services, such as screenings, and delaying a probable diagnosis, they could avoid learning about a premature death. “*And when I think about it, knowing means we have awareness, but we would rather not know. Our community members would rather not know and die, than know that they are sick”* (Ethiopian FGD 2). Community members’ anxiety of engaging with the healthcare environment, which they saw as communicable and potentially contagious, was also stated by the participants. *“They think if they go to the hospital, they will end up contracting a disease at the hospital. I hear that type of thinking from a lot of people. They believe what they do not know will not hurt them, so they resist going to the hospital unless they are deathly ill.”* (Ethiopian FGD 1). Participants from all three communities shared the belief that not knowing about and living in denial about having a disease is preferable to living with and suffering from one. *“People sometimes may not go to the screenings because they do not want to figure out or to discover if they have a disease. A lot of people would rather not know if they are sick or not.”* (38 year old, male, Somali, student).

#### 3.1.3. Fear of Western Medicine

Additionally, our findings suggest that avoidance of preventative health care is a result not only fear of disease, but also due to fear or mistrust of western medicine. According to participants, there is a significant distrust of US healthcare and services among ethnic Somalis, Ethiopians, and Eritreans. Participants expressed concern about maltreatment by healthcare practitioners, as well as a notion that western medicine is more dangerous and expensive than it is beneficial. One participant from the Somali community mentioned a fear of treatments such as invasive surgeries because they were afraid the doctor would harvest their organs instead of treating them. *“And even so, when after coming to the doctor’s visit the doctor prescribes like invasive treatments like surgery, they simply decline it. Because they’re so afraid of surgeries and they lack trust with the doctors. There’s a belief that they say the doctors actually out there to harvest our organs.”* (58 year old male, Somali, social worker/community activist). Another participant stated that senior members of the Somali community are refusing to take their prescribed medications out of fear becoming ill and dying as a result of the drug. “*And there’s a perception out there, they perceive and discuss amongst each other not to take the doctor’s medicine. Because what they believe is the doctors are out there to eliminate them. And I have seen a lot of diabetic patients and some with high blood pressure and cholesterol who are not taking their medications. They believe that this medication is going to kill them*.” (63 year old male, Somali, educator). Participants have stated that because of the fear of being mistreated, when an illness first starts to manifest, some community members are inclined to first use cultural or traditional medicines and delay seeking western medical care. Overall, participants acknowledged that when individuals from these communities access healthcare, it is often in the later stages of illness. Subsequently, when people eventually seek western medical care they may already be in a poor state of health and when they pass away, it may perpetuate the belief that seeking western medical care can lead to death.

### 3.2. Religious Beliefs/Views on Manifestation of Illness

Belief in religious institutions and trust in religious leaders play a major role in how community members access and utilize healthcare. Both Christian and Muslim participants indicated their health status can be ascribed to their religious efforts and God. *“First, people in our community have faith in God; we believe that diseases are naturally part of human life. And we believe that God heals, so we don’t really worry unless it interrupts our occupation or day to day life.”* (50 year old, male, Eritrean, nurse). Participants also mentioned turning to religious practices, such as spiritual remedies and holy water, along with traditional medicine before seeking western medical care. *“When people get sick, you will always see that they go try the traditional or spiritual remedies before they go see a doctor.”* (63 year old, male, Somali, educator). In particular, participants from the Eritrean and Somali communities mentioned that, ultimately, they have trust that God will care for them and will protect them in the face of adversity.

#### Predestination

Additionally, belief in predestination, that God has already decided one’s fate, has a major influence in preventative health care beliefs—if you are destined to become ill, then only with divine assistance can you remain healthy, and preventative care is superfluous. *“Our people have faith in God. Starting from me if I become sick, I believe that God will heal me. I put my full trust in God. You don’t need to go for health screening to make sure whether you are healthy or not. So many people trust on God for everything and use spiritual healing methods for any health problems”* (40 year old, female, Eritrean, housewife).

### 3.3. Immigrant Shared Experiences

We found that members of the Somali, Ethiopian, and Eritrean community’s experiences and beliefs are common to those of many immigrant communities; these experiences, in addition with cultural and religious beliefs, reinforce negative utilization of preventative healthcare. Three main subthemes related to this shared immigrant experience emerged: (1) preventative care is a low priority, (2) limited access to healthcare in their respective home countries, (3) intergenerational differences.

#### 3.3.1. Preventative Care Is a Low Priority

First, preventative care is a low priority among East African immigrants. In addition to providing for their family, learning a new language, and navigating a new life in the US, making time for seeking health care is not seen as a priority. Immigrants juggle multiple priorities simultaneously, and the type of jobs they hold may not permit them time off to seek either preventive or acute health care. Among the many competing priorities immigrants face, preventative care may be the least important one. *“You can’t access health care in a good way if you can’t risk getting fired if you take a day off to go get a wellness check. There are many examples like that.”* (32 year old, female, Somali, doctor).

#### 3.3.2. Attitudes toward and Uptake of Preventative Healthcare as an Immigrant or Limited Access to Healthcare in Home Country

Second, preventative care is not prioritized among people living *in* Somalia, Ethiopia, and Eritrea, and, furthermore, immigrants to the U.S. from these countries maintain this attitude. Participants from each community recounted visiting doctors in their home country only when there was a dire need for medical attention, *“Culture plays a very big role because nobody goes to the hospital unless they’re really sick. And that’s the culture they are coming back from home with.”* (58 year old, male, Somali, social worker/community activist). Participants also stated that accessing healthcare in their home countries is expensive and rarely sought out compared to individuals born in the U.S. *“The people who were born here, they are more likely to get screened whereas those who came here as immigrants with different cultures, they are more likely not to be screened. And the reason for that is the countries that these immigrants are originally from, usually health care and hospital in general are a few and rare*“ (38 year old, male, Somali, student).

#### 3.3.3. Intergenerational Differences

Additionally, participants discussed intergenerational differences between the older generations who immigrated to the US as adults and the younger generation who grew up with western medicine. The younger generation are taught to operate in a healthcare system that prioritizes prevention, *“People born in the USA have a culture of going to health providers regularly and doing health screenings. They have knowledge and understanding on the importance of going to a doctor and do health screenings. For instance, if a child becomes sick you call their doctor. They utilize it starting from their childhood. On the other hand, people who came from our country go to the doctor when they become sick only”* (38 year old, male, Eritrean, supervisor). As such, routine care and screenings amongst the younger generation are more common than the older generation. A participant who noted this difference stated that in the U.S. healthcare utilization starts from childhood and this behavior continues throughout adulthood, and, thus, these individuals are accultured better to western approaches to preventative health care attitudes. In contrast, for the older generation who immigrated to the U.S. at a later age, they have not assimilated to the western practices of preventative healthcare. *“Because the children that are born here are brought up within this same culture and health system. For example, they must have their immunizations current before they are even taken to school. On the contrary, their parents and everybody else is who from back home do not see health the same way they do.”* (38 year old, male, Somali, educator).

### 3.4. Structural Barriers Related to Health Systems

#### 3.4.1. Racism and Representation in Health Care

Participants expressed reluctance to seek health care services as a black immigrant because many were intimidated by numerous obstacles. Participants reported that not speaking English makes them feel like a burden, and there are additional cultural differences to contend with. There is also general sense of intimidation associated with entering settings where one’s race and/or ethnicity is not represented among health care providers and staff. Additionally, institutionalized and systematic racism makes it harder for black individuals to access high-quality health care despite their immigrant status. Some participants stated that they were subjected to unequal treatment in care and were prejudged based on their appearance or need for an interpreter. Thus, participants highlighted a need for providers representing their communities in clinical settings where they receive care in order to alleviate this problem and increase uptake of care.

#### 3.4.2. Lack of Culturally and Linguistically Appropriate Resources

Lack of resources, education and outreach regarding preventative health care has contributed to the poor preventative health care beliefs in these three communities. *“They wait until the disease has taken over you and the doctors cannot do anything about it. So, what I’m implying is, there needs to be an outreach for the community to educate them on the importance of early screenings and annual checkups.”* (58 year old, male, Somali, social worker/community activist). Language barriers and low health literacy regarding the US healthcare system have also led to avoidance of preventative care. Regarding low health literacy, participants discussed a need to understand the reasons for and importance of screening and other preventative care practices. Having a non-English language preference is not only a barrier in accessing resources but also an impediment in communicating with your provider. “*Sometimes when somebody wants to have services or the screenings done and they do not know the language through which to communicate to their doctors, is a big challenge.*” (54 year old, male, Somali, imam).

#### 3.4.3. Health Systems Barriers

Furthermore, understanding the policies and terms relating to the US healthcare system presents an additional challenge. Participants indicated both difficulty navigating the healthcare system and a lack of resources to help guide them through this complex system. Even individuals who are insured expressed confusion and difficulty navigating the healthcare system, particularly when it came to understanding what was included under their healthcare plan. “*One is language barrier. We do not know how to use insurance benefits even when we have health insurance. We do not know the system as much as those we are from America. It is a knowledge gap between both groups.”* (31 year old, female, Eritrean, case manager).

## 4. Discussion

In our findings we identified several overarching themes stemming from cultural and religious beliefs that go on to influence the preventative health care beliefs, attitudes and practices among the Somali, Ethiopian, and Eritrean immigrant communities in King County, WA. (1) Cultural beliefs, (2) religious beliefs and attitudes, (3) shared immigrant experiences, and (4) structural barriers related to health systems, collectively constitute significant barriers that prevent individuals from these communities from easily accessing or believing in the need for preventative care.

These data strongly suggest that community-centered and led programs that promote health literacy and preventative care, while addressing the attitudes and beliefs of East African immigrants, are required to effect meaningful change. When queried about what a successful intervention might look like, participants in all three groups said that religious leaders and institutions play an important role as gatekeepers in their community. These leaders and institutions have significant influence over their communities, both in terms of mitigating and perpetuating stigma. Interestingly, a Somali religious leader in a focus group/interview argued that Islam preaches about the importance of preventative care, suggesting that utilizing preventative care is an act of religious practice. This insight demonstrates how religious institutions and leaders may harness their intrinsic and unique advantages to highlight the need of preventative health care within their communities. 

We found that mistrust of westernized healthcare can act as a barrier in East African communities when seeking preventative healthcare. Fear caused by the lasting consequences of medical violence that Black, Indigenous and people of color (BIPOC) have been subjected to in western countries, lingers as symbolic violence that keeps African immigrants from accessing US healthcare. Lack of health insurance is also an obstacle among many immigrants in the U.S., despite the expansion of healthcare access granted by the Affordable Care Act, due to lack of or ineffective methods of enrollment [41]. Furthermore, we and others have found that insured individuals reported having problems navigating the healthcare system, particularly when it came to determining exactly what insurance benefits they were receiving [31]. Lack of information and inability to navigate the US healthcare system is a critical barrier to healthcare access for immigrants [42]. Our finding that language barriers and inadequate health literacy limited participants’ ability to communicate effectively with doctors and comprehend health information was also consistent with the results of other studies of immigrant health in the U.S. [31,42].

Some interventions developed for African immigrants have been designed to increase the uptake of preventative services, but have been primarily focused on one condition or outcome, for example Hepatitis B or cardiovascular disease [43]. Other researchers have delivered preventative health care in the context of community health fairs, which include a variety of services, typically a successful method of making services available to communities, especially among populations affected by structural barriers such as reduced access to insurance or language barriers [32,44]. These types of outreach efforts have been successful in many cases, and address multiple services that may benefit these communities. However, it may be necessary to address the cultural attitudes and beliefs that we observed in order to most effectively reach African immigrant populations with preventative care. Other studies have found similar themes when examining the uptake of health care among African immigrants, including lack of health insurance [41], difficulty navigating the healthcare system [31,42], and barriers related to language and inadequate health [31,42]. However, our study fills an important gap in the existing literature in that we focused on an uptake of preventive health care overall as opposed to the uptake of care or prevention services for one specific health problem, and we focused on three East African immigrant communities rather than one community.

East African immigrant groups value in-group identity, which is based on national, tribal, clan, linguistic, or religious associations and which typically emphasizes the differences between these communities. Despite these distinctions, our data indicates that these three communities share more similarities than differences when it comes to preventative healthcare behaviors. Somali, Ethiopian, and Eritrean immigrant communities in the United States have low uptake of preventative healthcare in part because their home countries did not emphasize it during the time the individuals were present there. Participants noted that in these East African countries, medical help was sought only when an individual was dangerously ill and traditional and religious treatments were ineffective.

Although our findings shed important light on various attitudes and beliefs that shape perception of need for and access to preventative health care services among African immigrants, our study has a few limitations. Our findings are most readily transferrable to other Somali, Eritrean, and Ethiopian communities in the U.S., but could possibly resonate with other African immigrant communities given the marked similarities and commonalities we identified across the three communities we worked with, though caution is advised in generalizing our findings to other African immigrant communities. The focus groups we conducted prioritized religious leaders from within these communities. For this reason, we may not have captured all community perspectives, but our interviews included a variety of community members hoping to capture a more dynamic range of perspectives. Lastly, the COVID-19 pandemic added complexity to recruitment for in-person interviews and for conducting the focus group discussions. In spite of these limitations, we believe our work is the most detailed to date involving the Somali, Eritrean, and Ethiopian communities around cultural beliefs and attitudes affecting preventative healthcare practices.

## 5. Conclusions

We discovered various cultural and religious beliefs that impact how some African immigrant communities in the United States approach preventative health. Cultural beliefs toward preventative care, mistrust of westernized healthcare, religious beliefs/views, shared immigrant experiences, and structural barriers to the U.S. healthcare system are all barriers to effective preventative care utilization. Addressing these norms and beliefs will be crucial in increasing preventative care uptake for African immigrant groups in the United States. Thus, community-based and led programs that promote health literacy and preventative care are critical, as are programs that address the attitudes and beliefs stated by our participants. Additionally, devoting resources to support increasing representation in healthcare, so that our healthcare providers reflect all the populations that they serve, would be an important step in the right direction to increase uptake of preventive care. In our future work, we hope to incorporate messaging around preventative health care into an HIV stigma reduction intervention led by religious and other community leaders in the Somali, Ethiopian, and Eritrean communities in King County, WA, with the goal of improving overall community health by increasing uptake of health screenings and vaccinations in these three communities.

## Figures and Tables

**Table 1:** Sociodemographic characteristics of study participants.

	Total Participants	Key Informant Interviews (KIIs)	Focus Group Discussions (FGDs)
	Number (*n* = 72) *	Number (*n* = 30)	Number (*n* = 43)
Age			
<30	5 (7%)	2 (7%)	3 (7%)
30–49	48 (66%)	16 (53%)	32 (74%)
50+	20 (27%)	12 (40%)	8 (19%)
Gender			
Male	37 (52%)	16 (53%)	22 (51%)
Female	35 (48%)	14 (47%)	21 (49%)
Country of birth			
Ethiopia	27 (37%)	11 (37%)	16 (37%)
Eritrea	17 (23%)	8 (27%)	9 (21%)
Somalia	27 (37%)	9 (30%)	18 (42%)
Kenya	1 (1%)	1 (3%)	0
U.S.	1 (1%)	1 (3%)	0
Language of interview			
Amharic	25 (34%)	9 (30%)	16 (37%)
Somali	27 (37%)	9 (30%)	18 (42%)
Tigrinya	18 (25%)	9 (30%)	9 (21%)
English	2 (3%)	2 (7%)	0
Kiswahili	1 (1%)	1 (3%)	0
Occupation			
Healthcare professional	15 (21%)	8 (27%)	7 (16%)
Religious leader	14 (19%)	4 (13%)	10 (23%)
Business/management	11 (15%)	5 (17%)	6 (14%)
Education/student	10 (14%)	5 (17)	5 12%)
Homemaker	6 (8%)	1 (3%)	5 (12%)
Laborer	3 (4%)	1 (3%)	2 (5%)
Not Working	3 (4%)	2 (7%)	1 (2%)
Other community leader	2 (3%)	2 (7%)	0
Other	9 (12%)	2 (7%)	7 (15%)
Community			
Ethiopian	27 (37%)	11 (37%)	16 (37%)
Eritrean	18 (25%)	9 (30%)	9 (21%)
Somali	27 (37%)	9 (30%)	18 (42%)
Kenyan	1 (1%)	1 (3%)	0
PLWHIV ^i^			
Yes	5 (7%)	5 (17%)	N/A
No	25 (34%)	25 (83%)	N/A
Religious Affiliation			
Orthodox Christian	12 (16%)	N/A	12 (28%)
Evangelical Christian	9 (12%)	N/A	9 (21%)
Islam	20 (27%)	N/A	20 (47%)
Catholic	1 (1%)	N/A	1 (2%)
Protestant	1 (1%)	N/A	1 (2)%

* One individual from the Ethiopian community participated in both a KII and a FGD. **^i^** PLWHIV People living with human immunodeficiency virus. Adapted from ref. [40].

**Table 2:** Main themes, subthemes, and example quotes for factors influencing preventative care beliefs and attitudes among Ethiopian, Somali, and Eritrean immigrant communities in King County, WA.

Main Theme	Subtheme	Example Quotes
Cultural beliefs and attitudes	Seeking health care only when in dire need	“Interviewer: What types of health screenings do people in your community have access to? Interviewee: There should be something that pushes you to go for health screening. I mean if I don’t have any feeling of sickness it don’t motivated to go to the doctor for a health screening. The only way to go to the doctor is when you feel sick.” (38 year old, male, Eritrean, supervisor)
		“I’ve met so many people from my community and this is what you need to understand. Culture plays a very big role because nobody goes to the hospital unless they’re really sick. And that’s the culture they are coming back from home with. When you come to this country there’s something called the annual checkups. People go to the doctors to have their checkups blood drawn and the importance for that I think is that if there’s anything unusual in your tests, they can be detected early enough, and they are able to treat that problem early. So, when someone misses the annual checkups, members of my community I’m afraid might only go to the doctor when there’s nothing that can be done about this situation. Meaning that the disease or the ailment has reached a level that cannot be treated. And this is first-hand information something that I’ve seen within a good number of my community members. These are people who have gotten cancer, hypotension, and all of this kind of thing. If they went to the doctors early enough, they might have been interventions. But unfortunately, they always go in the last stage or when it’s too late. So why is that? I think the reason for that is as I said earlier, culture.” (58 year old, male, Somali, social worker/community activist)
		“People like me who have diabetes the doctors told me that I needed once every six months. In Somalia, the culture was different, so we only went to the doctor when we were sick. Indeed, the health care system in the United States is very good but our community needs more education and outreach in accessing it. You will see a lot of people who might have health insurance is but do not have family doctors. The children mainly, one of the most important preventive care that you can give him is the immunizations. In the public-school systems, we are the second least immunized community. Many of our children do not have immunizations. For example, most of the Spanish children in this public-school system do not have immunizations because they don’t have medical insurance because they don’t have papers. On the contrary, most of Somalis have medical insurances but they still do not get preventive care. I don’t think many families take advantage of the health care system here. You see many of my friends who are taxi drivers or Uber drivers who haven’t seen their doctors for 5 or 6 years. They say they only go to the doctor when they have somewhere like a tooth, or an eye is hurting. For the most part, they don’t even have medical family doctors. Now that there are increasingly many Somali doctors here, the situation seems to be changing for the better a little bit. Even though they are many supported doctors and health professionals that do outreach in the community, he still the numbers are not as great as they were supposed to be when it comes to preventive care.” (63 year old, male, Somali, educator)
		“I don’t think much has changed. You know culturally, we’re good at seeking the solutions once there is a problem. But we’re not great at preventive care. For example, when there’s an outbreak and you tell people to go get preventive care, they won’t. They only go to the hospital after they got sick and you will see a lot of elderly populations like my age, 60 to 70 years of age, when they were told to take the flu shots, for example, most of them believe that the flu shots itself are the sickness. But you will see when they get sick, they will be down for 4 to 5 months at times. Preventive care is poor in our community. It’s mainly because of the culture and we are not used to these preventive measures. In Somalia, there were no studies and that’s why we are very poor in preventive care when it comes to the United States health system.” (63 year old, male, Somali, educator)
		“There is a little bit of difference between the Somalis that are born here and the ones that came from back home, you must agree. But that difference is not it’s not a big gap and this is why. But the parents themselves need to do more work because for example my son is 20 years old, he goes to college, but it’s still when it comes to routine checkups and his health, I must constantly remind him. But the problem becomes there are a lot of parents who were never exposed to the education system and who do not understand the importance of sending their kids to the checkups. In most cases, the kids might go to the hospital but then there’s nobody adult or none of their parents are accompanying them. They are some headway that’s been made, especially in immunizations that is the numbers have been improving significantly. But there are other problems still that needs to be addressed. For example, I see in the schools of children come in with asthma and I believe this and such. The problem is the parents do not give the medications to the children as they were supposed to. It breaks my heart when I see a child who is type one diabetic who has not been taking his medication because of the parents. Also, there are other medications that children need to take because of their special needs. But some parents would tell you that they would rather not give him the medication because it makes them gain weight or affect their mental development or make them hyperactive. And you will clearly see even the parents who became parents in this country and the ones who became parents back home are still connected in ways of not prioritizing the child’s health. Somalis are known to be 70% pastoralists and nomads. That means 80% of the time they’re moving from one place to another hence no need for a doctor visit. I think there are some components of that nomadic culture that still left in our heads even here.” (63 year old, male, Somali, educator)
		“Most of the people do something called, “bukaan-socod”, (walking-sick), or taking medicine like pain killers on the go and not really seeing a doctor unless they really need to. They might never go to a hospital. And that is the culture and how they were raised, no one should go to a hospital unless they are extremely sick. Whereas the ones here are so different because they are raised in such a culture that they are accustomed to being taken to the hospital often. They grow up with regular checkups with their doctors even at an early age.” (38 year old, male, Somali, student)
		“Generally, people in our community don’t go to the doctor when they’re feeling fine. They only go to the hospital when they’re sick or have a lot of symptoms. Health maintenance and you know preventive care that is just those are not a concept that exists within our culture.” (38 year old, male, Somali, educator)
		“I believe it’s a community-wide culture. I don’t think it is just a specific or community member of the whole community that has that culture. Because when I was little I used to hear of very popular saying within our communities at the time that loosely translates to, our people do not notice when the disease is it the goats but only notice it when it’s in the camels. So, people do not go to the doctor when they’re fine or when their symptoms are very early but instead, they wait until they are very sick. And that can cause people to have bigger problems, for example, they might go to the doctor when their disease has moved beyond the points after treatability.” (38 year old, male, Somali, educator)
	Fear of being ill/denial of illness	“There could be some denials within them when it comes to but they need help. And that itself is a barrier. When someone has let’s say diabetes, and they are in denial, or do not understand the depth of the problems it can cost them if they don’t take care of it. So, I can say the denial it’s a barrier to so many visuals in our community. Some of them are barriers to themselves too of not understanding. They might understand but they are still in denial of the fact.” (42 year old, female, Somali, health coach/interpreter)
		“I would like to say something about what happened recently. I was talking to a couple of guys and I receive a message from the Ethiopian Community Center, and it was about free testing for COVID-19 virus. And I said to them, “if you are going to the Ethiopian Community, you should get tested while you were there”. Their response was, absolutely not! We would rather die without knowing in 14 days than being stressed out about the test result. And all four of them did not want to go “in one voice”. And when I think about it, knowing means, we have awareness, but instead of transmitting it to someone else, we would rather not know. Our community members would rather not know and die, than knowing that they are sick” (Ethiopian FGD 2)
		“They believe what they do not know will not hurt them, so they resist going to the hospital unless they are deathly ill. I think we need to educate our community about early prevention and testing. In addition, we need to educate them the advantage of early testing to avoid being critically ill from preventative illnesses.” (Ethiopian FGD 1)
	Mistrust of western medicine	We only go to the doctor when we are sick, and in that case, that mentality is already out there. And even so, when after coming to the doctor’s visit the doctor prescribes like invasive treatments like surgery, they simply decline it. Because they’re so afraid of surgeries and they lack trust with the doctors. There’s a belief that they say the doctors actually out there to harvest our organs. And even though the doctor might tell them that if you decline the surgery you would come back here maybe worse than you are right now, they still declined. (58 year old, male, Somali, social worker/community activist)
		“I run an Islamic center where the community comes and prays and there are a lot of elderly people that come to that faith center. And there’s a perception out there, they perceive and discuss amongst each other not to take the doctor’s medicine. Because what they believe is, the doctors are out there to eliminate them. And I have seen a lot of diabetic patients and some with high blood pressure and cholesterol who are not taking their medications. They believe that this medication is going to kill them. So, there’s a lot of misinformation out there and it can affect a lot. “ (58 year old, male, Somali, social worker/community activist)
		“From what I understand and the question, a lot of our community members unless they are severely ill and bed-bound, when it comes to preventative care and testing, they are not willing to go to the hospital. They think if they go to the hospital, they will end up contracting a disease at the hospital. I hear that type of thinking from a lot of people. (Ethiopian FGD 1)
Religious beliefs and views on manifestation	Health status is ascribed to God	“First it is faith. It is based on faith. You believe that GOD don’t make you sick. Although going to a doctor can prevent it, they put their trust on God above all for prevention.” (53 year old, male, Eritrean, community leader)
		“Our People have faith in God. Starting from me if I become sick I believe that God will heal me. I put my full trust in God. You don’t need to go for health screening to make sure that whether you are healthy or not. So many people trust on God for everything and use spiritual healing methods for any health problems.” (40 year old, female, Eritrean, housewife)
		“They say only God can treat us and that might be true. And we often see so many people in our community the go back to the doctor when they’re really worse than they were.” (58 year old, male, Somali, social worker/community activist)
		“First, people in our community have faith in God; we believe that diseases are naturally part of human life. And we believe that God heals so we don’t really worry unless it interrupts our occupation or day to day life. However, if it interrupts our lives, first we traditional medications. We’re forced to go to modern clinics/hospitals only if that doesn’t work. That’s the general background of the people; like I said, you don’t resort to clinics unless you can’t function in your daily life. You prefer to resist the pain and continue.” (50 year old, male, Eritrean, nurse)
	Use of religious practices and traditional medicine to health	“For instance, when we were in Ethiopia we were not used to an annual checkup. We only see a physician when we are sick. We were first inclined to use traditional medicines such as herbs. We were never used to going to a physician thinking it is unnecessary and involves a high cost. Our community looks at modern medicine as harmful. I am not talking about those who are educated and have the information but about those who lack education and information.” (34 year old, female, Ethiopian, nurse)
		“Islam encourages prevention rather than the cure. If I could go back to the HIV questions again and talk about it in the way our religion does. Our religion tells us that if you want to get married to someone you have a right for you for both of you to get tested before you get married.” (54 year old, male, Somali, imam)
		“Even though people are encouraged to trust in God and believe in fate, it’s also important that they protect themselves from being unhealthy. In our religion Muslims are encouraged to use caution and apply effort to maintain their health. For example, one of the stories from the Prophet may peace be upon him, he used to run down when he was coming down a hill. And when his companions asked him, he said even though he believes in God’s will, it’s also important that we exert effort to ensure our own safety. Which means to take safety measures and precautions for our health. The religion encourages very much to work hard on one’s health and safety. Therefore, it’s very important to protect yourself and prevent diseases before they hit you compared to receiving treatment for a disease.” (56 year old, male, Somali, imam/religious leader)
		“Mental health by itself is highly stigmatized. Back in Ethiopia mental health is not discussed openly. It is usually assumed it is the result of bad spirits. So, people don’t go to western medicine but rely on prayer and holy water. If this is combined with HIV, people will say he is possessed with the devil and it’s because of his/her sins. This defiantly creates another level of stigma.” (34 year old, female, Ethiopian, nurse)
	Predestination; destined to become ill	“Muslims believe that everyone it is bound by fate and God’s destiny for them. No one is immune to fate. And that entails that most limits believe that all your feet have been preordained when you were in your mother’s womb. But they are also causes. So, people need to understand that this is just one of fate. It is one of the first six pillars of faith to believe in the “Qadr”, which means that everything happens because of God’s will. (63 year old, male, Somali, educator)
		“We think doctors can cure but not prevent illness. So, we only go to doctors when we become sick.” (38 year old, male, Eritrean, supervisor)
		“Cancer, they believe is a disease that one can get because of God’s will for you. And they believe unlike cancer people get HIV and AIDS because of their actions. That’s what society and the people in the community believe. But I believe both diseases can be God’s will for a person. “ (40 year old, female, Somali, community health worker)
Immigrant shared experiences	Preventative care is low priority	“You can’t access health care in a good way if you can’t risk getting fired if you take a day off to go get a wellness check. There are many examples like that.” (32 year old, female, Somali, doctor)
		“We only go to doctors when we become sick. For me, I prefer going to work rather than to go for health screenings.” (38 year old, male, Eritrean, supervisor)
		“You prefer to resist the pain and continue. I don’t think it’s the lack of trust of the modern health system but it might be due to some issues in insurance and economic level because modern clinics/hospitals require money. Or because you don’t have enough money or you don’t have insurance through your employment, you can’t pay out of your pocket so you prefer to work rather than spending money while you can still bear the pain… it’s like driving your car until it stops. You keep working until you’re broken; then you turn to God or government.” (50 year old, male, Eritrean, nurse)
	Limited access to healthcare in home countries	“The general understanding about accessing health facilities among Eritreans, Ethiopians and I know a few Sudanese is that what I observe is that we don’t go to clinics or hospitals unless we are sick. This is including myself. The thing is we must feel something abnormal or get seriously sick in order for us to see a doctor. I don’t people do yearly general checkup, prevention for instance checkups related to aging exposure to some decline or preventative screening are culturally unthinkable. One main reason is I think the idea that you don’t need to see a doctor unless you’re sick. In our culture, we go to a clinic/hospital only if one is very sick. Even when we’re sick, including myself, the illness must interfere with your daily life for you to see a doctor. Otherwise, if you can bear the pain while working and doing your daily life, we don’t bother to see a doctor.” (50 year old, male, Eritrean, nurse)
		“I believe we have not had the habit of checking up our health regularly. Otherwise, there is adequate health service in America and the facilities are well equipped. In general, my experience with these services was good.” (40 year old, female, Eritrean, housewife)
		“I think it is the mindset of thinking that I do not need it unless they feel symptoms or feel something different in their body. And I think a big part, or at least for people in my community, a big part has to do, most people the way they grow up. They may not have access to any healthcare and even if they did they might have only gone there if only there are in critical condition or someone they know. So, it is not normal to go to a hospital for a checkup or screening. So that may be just how they are growing up and also lack of access to those kinds of diseases and risk that comes with not doing a regular checkup. “ (22 year old, male, Eritrean student)
		“People born in the USA have a culture of going to health providers regularly and doing health screenings. They have knowledge and understanding on the importance of going to doctor and do health screenings. For instance, if a child becomes sick ask you to call their doctor. They utilize it starting from their childhood.” (38 year old, male, Eritrean, supervisor)
		I don’t want to overgeneralize the whole population and say that their own care for preventive health. That is how the culture was back home. People might change when they come here. And even after coming here, some people know communities still have the beliefs of the culture that they had back home. But also, there are some parts of our community that are up to date with the current culture and do things differently. But they’re also those who still go to the doctor only if they’re sick.” (38 year old, male, Somali, educator)
		“The children that are born here are brought up within this same culture and health system. For example, they must have their immunizations current before they are even taken to school. On the contrary, their parents and everybody else is from back home do not see health the same way they do.” (38 year old, male, Somali, educator)
		“The people who were born here, they are more likely to get screened whereas those who came here as immigrants with different cultures, they are more likely not to be screened. And the reason for that is the countries that these immigrants are originally from, usually health care and hospital in general are a few and rare. Therefore, not everyone gets a chance to get screened or see a doctor if they absolutely not need it.” (38 year old, male, Somali, student)
		“The people who were born here, they are more likely to get screened whereas those who came here as immigrants with different cultures, they are more likely not to be screened. And the reason for that is the countries that these immigrants are originally from, usually health care and hospital in general are a few and rare.” (38 year old, male, Somali, student)
Structural barriers related to health systems	Racism and representation in health care	“I think it is something that prevents a lot of people from accessing health care in the way that they would want to. There are huge cultural issues. There’s a huge intimidation accessing a space when people don’t look like you or don’t speak your language and you are a burden. So, there is a lot of that, that continues until today and with each community, they face unique challenges and the system like I said still has institutionalized and structural racism. Which makes it difficult for people who have intersectional identities to access.” (32-year-old female, Somali, doctor)
		“Institutional racism also shows up in how you get or receive health care it’s possible that all this data and research that the health professionals are basing their decisions on has been done on people who are not like us who do not look like us who are not limited in terms of resources and education as we are. With someone from our community goes to the hospital instead of getting the same treatment, sometimes it’s possible that they’re told since you’ve been through a lot more difficult situations; you don’t need to be treated for so and so illness. Because you’re already prejudged by how you look or what language you speak or do not speak.” (Somali FGD 1)
		“[In regards to planning a HIV testing project] I think the first thing would be to assemble a team that is diverse in a sense that, maybe some, a group of people that grew up here (Seattle/USA) and also people who came from Eritrea. People that speak English and also Tigrinya. As far as gender, equal representation of gender and experience in life. I would also find a way to have someone who is willing to participate that has HIV, that would be the first thing I would do. And then what is the next one?” (22 year old male, Eritrean, student)
	Lack of culturally and linguistically appropriate resources	“Well, as you know our people in our community are not aware enough. They go to hospitals and clinics when they feel really sick. They don’t do any checkups once in a while and I think this is really bad” (36 year old, male, Ethiopian, priest)
		“Interviewer: What do you think are the reasons the people do not take advantage of the health care system? Participant: It’s because of two reasons. The first one is the culture does not have used for preventive measures. The second one is a lack of education. People are poorly educated and to make matters worse we don’t even follow what the doctors tell us. I think that’s the main reason. For example, when people get sick, you will always see that they go try the traditional or spiritual remedies before they go see a doctor. Even though the spiritual healing it’s important at times but medicine also works. I think that’s the main course because people are not taking full advantage of the health care system.” (63 year old, male, Somali, educator)
		“Lack of education. There simply isn’t enough outreach for the community to educate them on the importance of annual checkups, an early intervention to do things about their situation before it’s too late. And in our prayer gatherings, I tell my community that they should go have their checkups and screenings so that they don’t wait for the interest situations to get out of hand. They wait until the disease has taken over you and the doctors cannot do anything about it. So, what I’m implying is, there needs to be an outreach for the community to educate them on the importance of early screenings and annual checkups.” (58 year old, male, Somali, social worker/community activist)
		“If you have the necessary information, you are more likely to explore the healthcare services. To give you a simple example, if I have nausea drinking peppermint tea sometimes might help shrink stomach sphincter and reduce nausea. I know this because I am a healthcare provider myself. So, information is power. People who do not have the necessary information about healthcare are more likely not to use the system.” (34 year old, female, Ethiopian, nurse)
		“It could be because of few reasons. People do not understand the importance of having preventive care. People sometimes may not go to the screenings because they do not want to figure out or to discover if they have a disease. A lot of people would rather not know if they are sick or not.” (38 year old, male, Somali, student)
		“Sometimes when somebody wants to have services or the screenings done and they do not know the language through which to communicate to their doctors, is a big challenge.” (54 year old, male, Somali, imam)
		“First there is language barrier. They are not sure whether the interpreter interpreted what they want appropriately or not. Second, the individuals also have their own problems. People who don’t have health insurance are more than those who have it. They don’t have deep knowledge on the importance of health insurance. At the same time there is nobody who can teach them the importance of health insurance.” (38 year old, male, Eritrean, supervisor)
		“Yes there is a lot. One is language barrier. We do not know how to use insurance benefits even when we have health insurance. We do not know the system as much as those we are from America. It is a knowledge gap between both groups. First of all, one has to convince himself to get screened and get services like case management and language interpretation services. It needs confidence and trust with case managers and develop courage and confidence. Even when you are not educated.” (31 year old, female, Eritrean, case manager)

## Data Availability

The corresponding author will allow sharing of de-identified data, data codebook, and other data elements upon request and ethics approval, and upon submission of relevant documents, such as research protocol, and signed data access agreement.

## Supplementary Materials

The following are available online at https://www.mdpi.com/article/10.3390/ijerph182312706/s1, File S1: The Harambee! 2.0 KII and FGD guides.
